# Response in Ambient Low Temperature Plasma Ionization Compared to Electrospray and Atmospheric Pressure Chemical Ionization for Mass Spectrometry

**DOI:** 10.1155/2018/5647536

**Published:** 2018-12-18

**Authors:** Andreas Kiontke, Susan Billig, Claudia Birkemeyer

**Affiliations:** Research Group of Mass Spectrometry at the Faculty of Chemistry and Mineralogy, University of Leipzig, Linnéstr. 3, 04103 Leipzig, Germany

## Abstract

Modern technical evolution made mass spectrometry (MS) an absolute must for analytical chemistry in terms of application range, detection limits and speed. When it comes to mass spectrometric detection, one of the critical steps is to ionize the analyte and bring it into the gas phase. Several ionization techniques were developed for this purpose among which electrospray ionization (ESI) and atmospheric pressure chemical ionization (APCI) are two of the most frequently applied atmospheric pressure methods to ionize target compounds from liquid matrices or solutions. Moreover, recent efforts in the emerging field of “ambient” MS enable the applicability of newly developed atmospheric pressure techniques to solid matrices, greatly simplifying the analysis of samples with MS and anticipating, to ease the required or even leave out any sample preparation and enable analysis at ambient conditions, outside the instrument itself. These developments greatly extend the range of applications of modern mass spectrometry (MS). Ambient methods comprise many techniques; a particular prominent group is, however, the plasma-based methods. Although ambient MS is a rather new field of research, the interest in further developing the corresponding techniques and enhancing their performance is very strong due to their simplicity and often low cost of manufacturing. A precondition for improving the performance of such ion sources is a profound understanding how ionization works and which parameters determine signal response. Therefore, we review relevant compound characteristics for ionization with the two traditional methods ESI and APCI and compare those with one of the most frequently employed representatives of the plasma-based methods, i.e., low temperature plasma ionization. We present a detailed analysis in which compound characteristics are most beneficial for the response of aromatic nitrogen-containing compounds with these three methods and provide evidence that desorption characteristics appear to have the main common, general impact on signal response. In conclusion, our report provides a very useful resource to the optimization of instrumental conditions with respect to most important requirements of the three ionization techniques and, at the same time, for future developments in the field of ambient ionization.

## 1. Introduction

In the recent past, the interest in multiselective methods analyzing complex samples as quick as possible, and its components as sensitive and complete as possible, has grown tremendously. Thus, the era of the “omic” techniques evolved and a growing number of scientists are facing now the very challenging task to set up analytical methods that would be applicable to as many target compounds as possible at a time, in very different matrices. This task demands methods with a very high performance in terms of analytical resolution, selectivity and sensitivity. Therefore for this purpose, high-performance analytical detection methods such as mass spectrometry (MS) are very useful. MS is widely used for many multiselective techniques, the nowadays so-called “omic” techniques such as proteomics [1], metabolomics [2], or lipidomics [3], as to name a few. In forensics [4], drug development [5] and process monitoring [6], structural elucidation of natural substances [7], and even in the identification of counterfeits [8], MS is also the method of choice because of the rich information this technique delivers from a sample.

For MS analysis, the target molecule is converted into an ion, which subsequently needs to be transferred into the gas phase to enter the analyzer for determination of its* m/z*, mass-to-charge ratio. The basic principle of mass spectrometers remained almost unchanged in recent years. For example for the separation of the ions, MS analyzers such as the linear ion trap [9], the reflector TOF (time of flight) [10] and the FT-ICR (Fourier-transform ion cyclotron resonance) [11] were already established decades ago, and the introduction of the orbitrap [12] based on the work of Kingdon [13] and others, can be considered as the latest remarkable step forward. Nonetheless, the technical quality of the devices improved constantly in recent decades leading to a significantly enhanced performance of modern instruments not only in terms of sensitivity but also in that high resolution instruments became increasingly common in analytical labs.

Therefore, the main task of instrumental development nowadays seems to broaden the applicability of the method and, thus, the ionization process, in which end the target species is ionized and brought to the gas phase, became the greatest limitation of MS. However, analytical questions with their corresponding target compounds are highly diverse and the anticipated target analytes have very different prerequisites for ionization, which is why there are many different methods used. In detail, ionization requirements of small volatile molecules differ a lot from those of nonvolatile and large molecules such as proteins, and the polarity also plays a crucial role. Based on the energy that is transferred to the analyte during the ionization process, a classification into “hard” and “soft” ionization can be made [14]. Furthermore, the ionization types can be divided into vacuum methods and those under atmospheric pressure, or the order of ionization and desorption to the gas phase, i.e., if ions or neutrals are brought to the gas phase. In particular, the introduction of atmospheric pressure ionization (API) techniques can be considered a quantum leap within this context. Among API methods, electrospray ionization (ESI-MS) has become one of the most commonly employed techniques in analytical chemistry, mainly due to its broad applicability to polar and semipolar compounds and the superior selectivity which is achieved in combination with high resolution separation techniques such as liquid chromatography or capillary electrophoresis [15]. Another common API technique is the atmospheric pressure chemical ionization (APCI).

With the introduction of two ambient ionization techniques for API-MS, DESI (desorption electrospray ionization) [16] and DART (direct analysis in real time) [17], direct analysis of samples with minimal or no sample preparation became possible offering an enormous potential for saving time and resources. The introduction of ambient ionization at atmospheric pressure for mass spectrometry (AI-MS) attracted the interest of many researchers in the field and various ionization techniques have been described in recent years. Among those, plasma-based techniques including the low-temperature plasma probe (LTP) require very little resources thereby providing great potential for implementation in mobile analytical devices [18]. The ultimate objective of current research in that area is to increase the range of applicability of MS and therefore it is essential to understand the different ionization mechanisms in detail. However, systematic studies on relative signal response among the different API techniques, such as the influence of the analyte and matrix characteristics on relative signal intensity, are still rare. Here, we review the available literature to compare the two most commonly employed API methods, namely ESI and APCI, with LTPI. To enable a direct comparison of these techniques, we add our review with own data revealing analyte characteristics that make their carrier particularly suitable for either of the three methods. We hope that sharing our results will help to further improve the general understanding of different API mechanisms, its common requirements but also the selectivity of the different techniques.

## 2. ESI and APCI – Two of the Most Common Atmospheric Pressure Ionization Techniques

### 2.1. ESI Mechanism

**Figure 1:**
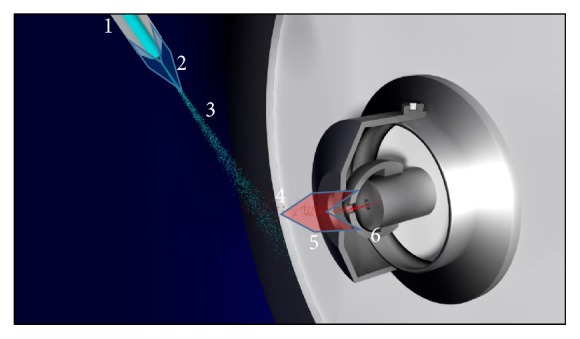
Schematic drawing of the ESI source in front of the MS inlet. 1, sprayer; 2, nebulizer gas (blue arrow); 3, spray/plume; 4, ions (red dots); 5, dry gas (red arrow); 6, MS inlet.

Dole et al. established the basics of ESI about 50 years ago [19, 20]. Years later, John Fenn's group, inspired by Dole's work, was able to show that using ESI, it was possible to make large, polar and even instable molecules accessible to MS [21, 22]. For ESI, the chargeability of the analyte is absolutely essential [23] which is achieved through different processes, whether by charge separation (e.g., deprotonation) or adduct formation (e.g., protonation) [24] or, less frequently, by electrolytic oxidation or reduction [25, 26]. A schematic view of an ESI-source is illustrated in Figure 1. First, the dissolved analyte is pumped through a conductive sprayer capillary. Flow rates here range from several nL/min (nanoESI) to the mL/min-range (conventional liquid chromatography, LC coupling with ESI). An electrical field (E ≈ 10^6^ V/m) is created by applying a voltage difference in the kV range (usually between 2 to 6 kV) between the sprayer capillary and the MS, which acts as a counter electrode. The respective charged species (positive or negative, depending on the applied direction of the electric field) are separated by acceleration to the MS. Depending on the vendor, either the sprayer capillary is grounded while the MS inlet is on high voltage (*needle on ground* configuration), or the sprayer capillary is on high voltage and the MS inlet on low voltage (*needle on potential*).

In the sprayer capillary, the first important process takes place, where the strong electric field leads to a charge separation by electrophoretic migration within the liquid. In the positive ion mode as an example, the cations are accelerated in direction of the MS inlet, while the anions are attracted to the inner capillary wall and can be oxidized there [27], which is reversed in negative ion mode. When the liquid sample leaves the sprayer capillary, the counteracting effect of the surface tension of the solvent on one, and the attracting force of the applied electrical field on the ions in the solution on the other side, is responsible for the formation of a cone, the so-called* Taylor-cone* named after Sir Geoffrey Taylor who was one of the first scientists describing and investigating this phenomenon [28]. The shape of the cone and thus the further formation of the so-called jet or filament, formed at the point of the highest charge density, the break-off of droplets from it and the resulting properties of these droplets all depend on the operating parameters of the mass spectrometer (e.g., needle voltage and flow rate) [29]. Basically, as soon as the surface tension of the solvent is exceeded by the electrostatic forces between the dissolved ions and the applied potential at the MS-entrance, the filament disintegrates and droplets are pinched off [30] due to instabilities and propagating waves along the filament [29]. This process is often supported by a nebulizer or sheath gas, an inert gas such as nitrogen, which encircles the ESI plume and thus diminishes the influence of surface tension. All of these droplets carry a net charge reversed to the MS electric pole. Subsequent evaporation of the solvent supported by a heated dry gas (nitrogen) causes the droplets to shrink. In succession, the charge density on the surfaces increases until the coulomb repulsion forces between the like-charged ions exceed the cohesive intermolecular forces at the so-called* Rayleigh* limit [31], where the surface tension equals the coulomb repulsion, and droplet fission occurs. The process of evaporation and splitting is repeated several times until the droplets have radii of a few nanometers.

Two theories are commonly accepted for gas-phase ion formation during the electrospray process, the* ion evaporation model* (IEM) [32] and the* charged residue model *(CRM) [19]. IEM applies mainly to low-molecular weight analytes and suggests that the resulting coulombic repulsion is strong enough to overcome surface tension and a dissolved ion is released from the droplet surface to the gas phase. It is believed that this mechanism takes place when the droplets have radii smaller than 10 nm, as the Rayleigh instability with droplet fission is preferred for larger radii [32]. The CRM on the other hand can be utilized to explain the release into the gas phase of larger analytes such as proteins [19], it is assumed that the cycles of shrinkage and fission caused by solvent evaporation ultimately end in a single ion in a solvent shell, which transfers the charge to the analyte after drying. Konermann et al. proposed an advanced model for gas phase ion formation called chain ejection model (CEM) [33]. It is assumed that this mechanism can be used for large molecules with nonpolar side chains, for example proteins that are unfolded due to an acidic solvent (e.g., a mobile phase in LC), while CRM can be used for native proteins [34]. The CEM describes an IEM-like process, in which the nonpolar side chain migrates to the droplet surface and from there is expelled into the gas phase till the protein separates from the droplet completely [33, 35].

### 2.2. APCI Mechanism

Atmospheric pressure chemical ionization, APCI, is the second most important ionization method when it comes to LC-MS coupling. It is a gas phase ionization technique in which the analytes are ionized similar to chemical ionization, with the difference that the ionization takes place at atmospheric pressure and not under reduced pressure. First, ^63^Ni was used in the ionization source [36, 37] but was soon replaced by a corona discharge since it produces comparable spectra [38] with an improved dynamic range [39], easier manufacture, use, maintenance and disposal with regard to radioactive waste. Approaches using a glow discharge were also described [40, 41].

**Figure 2:**
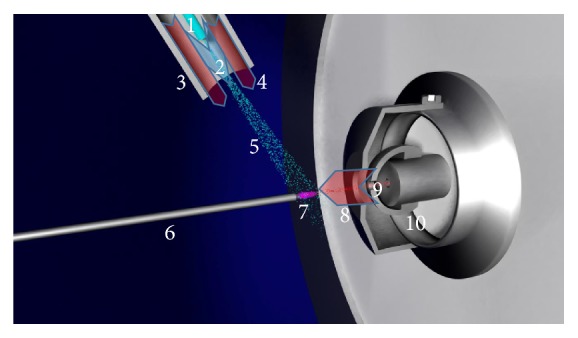
Schematic drawing of the APCI source in front of the MS inlet. 1: sprayer; 2: nebulizer gas (blue arrow); 3: heater; 4: auxiliary gas (red arrow); 5: vaporized spray; 6: corona needle; 7: corona discharge; 8: dry gas (red arrow); 9: ions (red dots); 10: MS inlet.

APCI is typically used for small molecules (<1000 u) that are not polar enough for efficient electrospray ionization (ESI). Although APCI was developed earlier than ESI and according to the literature, APCI is supposed to be less vulnerable to matrix effects [42], ESI has become much more widespread. Possibly, the reason for this was the great interest in the analysis of large proteins [43], which remained inaccessible to APCI. However, APCI benefited from the rapid development and expansion of ESI and the related development of atmospheric pressure interfaces, since in the late 1980s and early 1990s all major MS device manufacturers introduced APCI sources [44]. Most instruments can accommodate an ESI* and* an APCI source, since the two sources can be easily exchanged due to their similarity.  Figure 2 illustrates the general appearance of a typical APCI source.

Instead of a sprayer capillary with spray voltage, a pneumatic nebulizer with a downstream vaporizer or heater block is used here. Nitrogen is commonly used as nebulizing and auxiliary gas. Analyte and solvent are vaporized in the heater (up to 550°C) near the corona discharge needle. A high potential of 3-5 kV is applied to the needle since corona discharges generally occur at sharp-edged points or corners if the electric field is sufficiently large. Corona discharge is an uneven discharge; it acts as an electron source and the effects are produced at the electrode, i.e., a strong electric field, ionization, and the resulting glow [45]. The ionization mechanism has already been thoroughly investigated and the most important reactions of positive-mode APCI are described as follows [38, 46–49].

First, nitrogen is ionized by electrons generated by the corona discharge. The generated ion reacts with surrounding nitrogen and forms N_4_^ ^^+·^. Although the ionization energy of nitrogen is higher than that of the analyte or solvent, ionization of nitrogen is most likely due to its high concentration (nebulizing and auxiliary gas) compared to the analyte or solvent.

(1) N2+e-→N2 +·+2e-N2 +·+2N2→N4 +·+N2

The high-energy nitrogen ions N_2_^ ^^+·^ and N_4_^ ^^+·^ transfer the positive charge very quickly to the solvent or to water (as traces in surrounding gas), which is why the latter ion cannot be detected under standard conditions [38].

(2) N2 +·+S→N2+S+·N4 +·+S→2N2+S+·

Considering the higher concentration of solvent molecules compared to analyte molecules, S^+·^ is most likely to react with other solvent molecules by hydrogen abstraction leading to the formation of protonated solvent and solvent clusters.

(3) S+·+S→S+Hs++S-Hs·Sn-1+Hs++S→Sn+H+

Finally, the analyte can be ionized by proton transfer if the gas-phase basicity of the analyte is higher than that of the solvent (or solvent cluster) [49].

(4) $$\begin{array}{c} M+{\left[\;S_{n}+H\;\right]}^{+}\to {\left[\;M+H\;\right]}^{+}+S_{n} \end{array}$$

In addition to protonation via the solvent (cluster), there are also possibilities of ionizing the analyte as a radical cation [47]. Thus, the analyte can also be ionized directly by a high-energy electron, or by charge transfer from the high-energy nitrogen species and ionized solvent, respectively.

(5) M+e-→M+·+2e-M+N2 +·→M+·+N2M+S+·→M+·+S

Since ionization takes place at atmospheric pressure, excess energy is released by impacts with nitrogen (nebulizing and auxiliary gas) which makes APCI a softer ionization method than chemical ionization under vacuum (especially since the negative mode is even softer and is therefore particularly suitable for the determination of the molecular mass) [47]. However, due to the high-energy processes during ionization, some fragmentation might occur, which sometimes can also be helpful for structural elucidation.

### 2.3. Relative Response in ESI and APCI

One of the major drawbacks of the atmospheric pressure techniques is their rather selective sensitivity with respect to certain analyte's characteristics. For example, ESI response of equimolar concentrations of different analytes in solution can vary by > 3 orders of magnitude [50]. Response depends on all analyte's, solvent's, and instrument's characteristics influencing the processes of ionization and ion desorption, e.g., solution and gas-phase basicity and chemistry, polarity (log P), the number of charge sites or different charge states in solution (pH), the susceptibility to oxidation/reduction, the tertiary structure and molecular size of the analyte (mainly for higher molecular weight compounds), vaporization energy, or surface affinity [51–59]. However, the reported findings depended on study design: for example, Zhou and Cook [60] found that signal intensities for caffeine and arginine were independent on the pH of the solution as a consequence of their basic character; these bases stay protonated till a pH of 10 (pKa of caffeine). Ehrmann et al. [61] did not find evidence for the importance of gas-phase basicity; very likely, these results were again influenced by the strong basic character of the compounds under investigation. Thus, Kiontke et al. found that the general importance of the fundamental parameters as there were compound basicity, polarity, and molecular surface, respectively, hold true to be factors indeed determining ESI sensitivity; their quantitative impact, however, is rather subject to interplay with other parameters such as solvent pH and instrumental configuration [15].

One of the most important compound characteristics known to determine the intensity of the (M + H)^+^ signal in MS after electrospray ionization, is the extent of its protonation in solution, i.e., the solution basicity [61, 62]. The ability to attract a proton in solution is best described by the pKa of the respective compound that can be retrieved from public databases such as SciFinder and ChemAxon. Solution basicity is so closely related to the interplay of electron-donating and withdrawing effects in the structure of the analyte, i.e., the electron density of the investigated molecules, that these parameters cannot be separately assessed; ESI-response is determined to the same extent by solution basicity as by the structural effects that also account for the basicity of a compound [15].

The second important compound characteristic after basicity is compound polarity. The correlation between polarity and signal response is interacting with solvent pH* and* polarity which has not been extensively studied yet [61]. In the literature, different findings about this interaction were reported: polar analytes provided a higher relative ESI response at neutral pH, while nonpolar analytes appeared to be less sensitive to solvent pH [63], the log D for pH 10-14 was best correlated with ESI response at pH 7 [62], while Kiontke et al. [15] found the correlation of the response at pH 7 and 3 strongest with log D at pH 3, potentially related to the fact that in ESI positive mode electrochemical oxidation leads to acidification of the ESI solvent.

At acid pH, the polarity of the molecular surface becomes important but in dependency on the amount of the organic or aqueous, respectively, phase. Nonpolar targets particularly benefit from acidification of the aqueous solvent* only at low organic content*. However, when decreasing the solvent pH, ion suppression by pH modifiers has to be considered which occurs by impaired desolvation due to decreased solvent volatility. In return, the volatility of a compound exerts its influence particularly upon signal enhancement by acidification suggesting that it is an additional advantage in competition to a pH modifier, since this effect was independent from compound basicity itself [15].

As to the research about sensitivity in APCI, it has been comparably less investigated. Sunner et al. grouped the compounds with respect to APCI response into three different classes [49, 64, 65]. One class, mostly nitrogen bases, are compounds with a gas phase basicity > ~830 kJ/mol, which easily undergo gas-phase protonation. Concerning compounds with a gas-phase basicity below ~830 kJ/mol, for another group, often oxygen-containing bases, sensitivity increases with increasing basicity under thermodynamic control. The third group consists of substances forming gas-phase hydrates with a very low stability, which sensitivity is mostly influenced by the gas temperatures [49, 66–69]. Other, compound-independent parameter were suggested to be the reagent ion plasma density [70, 71], discharge current [72], space charge effects [73, 74], the residence time in the ion source [49], the distance between the discharge needle and the vacuum interface [72], and flow dynamics [75].

**Figure 3:**
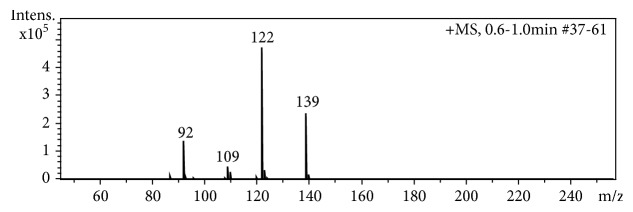
APCI mass spectrum of 10 *μ*M 4-nitroaniline in methanol/H_2_O 1:1 (*v/v*).* m/z* 139 is the molecular ion (M + H)^+^.

For a systematic comparison, we analyzed the relative signal response of the same 31 aromatic amino compounds that were analyzed with ESI [15] to assess the quantitative impact of the investigated molecular descriptors. Compared to ESI in agreement with [76], APCI produced more fragments and less sodium adducts. As an example, Figure 3 shows the APCI spectrum of 4-nitroaniline.

The APCI spectrum of 4-nitroaniline is dominated by four species of which* m/z *139 represents the [M + H]^+^, the first cleavage [M+H-17]^+^ most likely corresponds to the elimination of an OH-radical (confirmed by accurate mass,* m/z *= 122.0480) despite of suggestive NH_3_-cleavage. Oxygen can also be protonated in the gas phase and an O-protonated species is formed [77, 78]. The presence of* m/z *109 indicates a cleavage of NO, while* m/z* 92 corresponds to an HNO_2_-cleavage. This example illustrates the considerably harder ionization conditions of APCI compared to ESI.

**Figure 4:**
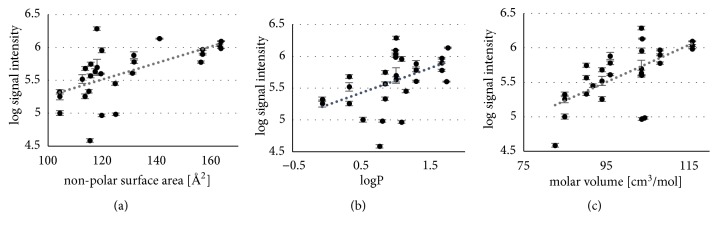
Log signal intensity (peak height) of APCI measurements in dependency on (a) the nonpolar surface area (SciFinder), (b) log P (ChemAxon), and (c) the molar volume.

The logarithmized signal intensities of APCI measurements were then analyzed for Pearson's correlation with the values of the available molecular descriptors. The results are presented in Figure 4 (note that correlation strength was evaluated according to [79] (a) very weak: 0.00-0.019, (b) weak: 0.20-0.39, (c) moderate: 0.40-0.59, (d) strong: 0.60-0.79, and (e) very strong: 0.80-1.0).

A moderate positive correlation was found for the polarity descriptors molecular nonpolar surface area (Figure 4(a), data from SciFinder, and R = 0.59, p < 0.01) and log P (Figure 4(b), data from ChemAxon, R = 0.48, p < 0.01), and a strong positive correlation was found for the molar volume (Figure 4(c), R = 0.64, p < 0.001). Curiously, sensitivity was not enhanced with increasing gas-phase basicity. Possibly, since most of our target compounds were nitrogen bases with a gas phase basicity above the claimed threshold of ~830 kJ/mol [49, 64, 65], the quantitative impact of gas-phase protonation is exhausted for our compounds and here, we observe the impact of other compound characteristics beyond that. However, concerning volatility, vaporization enthalpy also had no significant influence, which may be in agreement with Sunner et al. [49] who rather suggested the gas temperature to enhance signal response, indicating that* solvent* volatility may be more important than the* compound* volatility.

Thus, our results from analysis of the amines rather emphasize that desorption characteristics of the target compounds play an important role for this atmospheric pressure ionization technique. In conclusion, descriptors such as the molecular nonpolar surface area, log P and molar volume are very important for the surface activity of an analyte. The larger the nonpolar surface area and log P are, the higher the presence of the analytes at the liquid-air interface [23, 80] improving desorption, hence, ionization efficiency. In addition, the positive influence of the molar volume might attribute to the size of the molecule, which with increasing size not only stabilizes the protonated form in the gas phase [81] but also increases the probability of at least partial occupancy of the droplet exterior.

In summary, APCI appeared much less selective compared to ESI, and solution and gas-phase basicity, which are the determining compound characteristics in ESI, did not play the same crucial role for the compounds under investigation. Consequently, ion suppression in APCI-MS was often reported not as severe as in ESI-MS [82] which may be beneficial for methods employing less sample preparation steps. Instead, molecular polarity descriptors determining the surface affinity and desorption characteristics of an analyte, seem to play the major role here which in ESI become only more important mainly in situations where the protonation homeostasis is no longer limiting ionization, i.e., at low pH [15]. However, for the molar volume the existence of an optimal value is presumed since APCI is known less suitable for high molecular weight analytes >1500 u [83]; interestingly, ~1000 u was suggested as an approximate range for the change between the IEM to the CRM regime [84], which was suggested to be one of the reasons of this appearance [85]. 

## 3. Modern Ambient Ionization – Cold Atmospheric Plasma as an Easy and Particularly Promising Technique

### 3.1. Mechanism of Low Temperature Plasma Ionization

At the beginning of the 21st century, several new ionization techniques denoted as “ambient” were introduced beginning with* desorption electrospray ionization* (DESI) by Takats et al. 2004 [16] and* direct analysis in real time *(DART) by Cody et al. 2005 [17]. The term “ambient” introduced by Takats* et al*. for this type of ionization, is not strictly defined [86] and many similarities exist with the traditional methods APCI, APPI (atmospheric pressure photoionization) [87], AP-MALDI (atmospheric pressure matrix-assisted laser desorption/ionization) [88], or hybrid ionization techniques, for instance by coupling laser desorption to the traditional methods APCI [89–91] and ESI [92]. First of all, all operate at atmospheric pressure so that samples do not have to be introduced into a vacuum. Furthermore, in most of the ambient ionization methods, ESI- and APCI- processes are dominating with few limits to the new ambient methods. Since this is a very active field in MS research, various reviews already outlined requirements and possibilities of the ambient methods, e.g., Cooks et al. [93], Harris et al. [94], Weston [95], and others [96–98].

**Figure 5:**
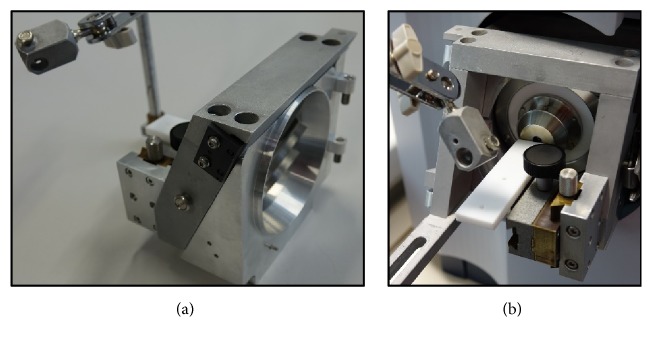
(a) Modular frame for mounting the plasma source to Bruker mass spectrometers and (b) completely mounted to a Bruker micrOTOF.

In contrast to the closed ionization chambers of traditional mass spectrometers, ionization with ambient methods occurs* outside* the instrument, so that the surface of very large or bulky objects can also be analysed. According to Harris et al. [94], ambient ion sources can be easily coupled to most differentially-pumped mass spectrometers, eventually with the help of special adapters as illustrated in Figure 5.

This feature promises easy implementation in mobile on-site MS-analysis [99, 100]. An enormous advantage and usually mentioned first when talking about AI-MS is the minimum or no sample preparation. This means that extraction, derivatization, desalting, dissolution, pre-concentration or separating techniques do not have to take place in advance, which can lead to enormous savings in time and resources. Furthermore, the ambient ionization methods should ionize at least as softly as the traditional atmospheric pressure methods and should also maintain the native state and spatial integrity of the sample. Since the ambient ionization methods are essentially noninvasive, they are ideally suited for the examination of sensitive surfaces such as living biological tissues which makes these techniques particular interesting for* in situ* analysis in clinical diagnostics and surgery.

Within the many ambient methods introduced so far, plasma-based ambient ionization techniques are particularly fascinating thanks to their simple and at the same time inexpensive, yet usually robust construction. They are not dependent on high purity solvents, they generate mainly easy-to-interpret mass spectra since the ionization mechanism typically involves the protonation of the analyte by protonated water clusters that are formed by the interaction of the plasma with atmospheric water from the ambient air [101, 102]. In literature, numerous variations of plasma ionization techniques can be found. Differences exist, for example, in the voltage applied to generate the plasma, DC is used for DART,* atmospheric-pressure glow discharge,* APGD [103], and the corresponding* flowing atmospheric-pressure afterglow,* FAPA [104], while AC is applied in* dielectric-barrier discharge ionization*, DBDI [105],* low temperature plasma ionization,* LTPI [106], and* plasma-assisted desorption/ionization,* PADI [107]. Also the techniques differ in temperature; some are operated without additional heating (PADI, LTPI, DBDI), with Joule heating (APGD, FAPA) or, in the case of DART, the temperature is increased by additional heating to assist thermal desorption [97].

**Figure 6:**
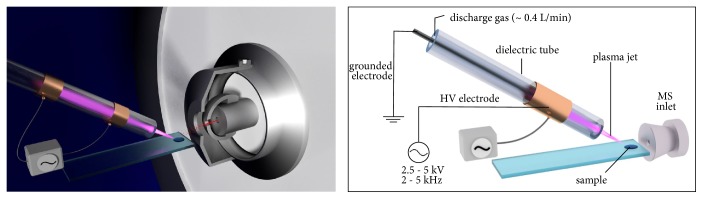
Physical appearance of the LTPI-source: schematic drawing of the plasma directed onto a target.

In comparison to all other techniques including DART, characteristic of the so-called* dielectric barrier discharge plasma* is that at least one electrode is covered with a dielectric, a nonconducting material (insulator, typically glass or ceramics) in which the charged particles or rather the charge cannot freely move in contrast to conductors. For this reason, DBD plasma must be operated with AC, as no further charge transport is possible with DC. Under AC, on the other hand, the dielectric acts as capacitor whose capacitance depends, among other things, on its thickness and permittivity [108].  Figure 6 illustrates the principal physical appearance of a DBD-LTPI source and its corresponding parts.

A dielectric barrier discharge (DBD) or “silent discharge” is a nonequilibrium plasma under atmospheric pressure [109]. The use of a dielectric between the electrodes and the plasma gas (e.g., helium) limits the current, resulting in nonequilibrium, low-temperature plasma (LTP) [109] enabling the direct ionization from a surface and subsequent MS analysis of compounds at a very low process gas flow rate, with high signal intensity and minimal fragmentation.

In LTP, statistically present electrons are accelerated and, impacting the surrounding gas, the electrons release their energy to collision partners producing more electrons, ions and excited species. If the particle density is low or the electric field is strong enough, the frequency of excitation is important because it determines the behaviour of electrons and ions. Due to their lower mass, the speed of the electrons on average will be higher than the speed of the gas molecules, atoms and/or ions. In this case it is called nonequilibrium plasma, or cold plasma [110]. If the particle density is so high that the mean free path of the electrons is small or the electric field is very low, the energy of the heavy gas particles will approach that of the electrons and all particles will have the same temperature. This is referred to as equilibrium plasma, called hot plasma.

The ionization mechanism was investigated in more detail for a helium-based LTP [101, 111] where the helium dimer He_2_^ ^^+·^ formed near the plasma discharge [112–114] was found to be the predominant positive ion for charge transport and formation of N_2_^ ^^+·^ through a charge transfer reaction, which, analogously to APCI, is then responsible for the formation of water clusters. N_2_^ ^^+·^ is mainly present in the afterglow region of the He-LTP. To a lesser extent, the ionization of N_2_ can also be caused by Penning ionization upon collision with excited helium. Other processes of formation are not further mentioned here due to their low probability.

(6) He2 +·+N2+e-→He2 ∗+N2 +·+e-He∗+N2→He+N2 +·+e-

The occurrence of N_2_^ ^^+·^ establishes a similarity with the APCI-mechanism. Indeed, with increasing concentration of N_2_ in the He-plasma, the formation of N_4_^ ^^+·^ increases due to conversion from N_2_^ ^^+·^.

(7) N2 +·+2N2→N4 +·+N2N2 +·+2N2+He→N4 +·+N2+He

Penning ionization of N_2_ and the charge transfer to N_2_^ ^^+·^ are relatively suppressed in an argon discharge, which on the other hand produces a strong OH response when analyzed with optical emission spectroscopy [115, 116]. The spectrum of the nitrogen plasma jet again has a series of NO_*γ*_ lines. Here, the N_2_^ ^^+·^ line is weaker than N_2_ second positive system bands, which is quite different from that of a helium jet.

N_2_^ ^^+^·^^ can transfer the charge to atmospheric water, which leads to protonated water clusters and finally to protonation of the sample [117].

(8) N2 +·+H2O→H2O+·+N2N4 +·+H2O→H2O+·+2N2H2O+·+H2O→H3O++OH·H3O++H2O+N2→H2O2+H++N2H2On-1+H++H2O→H2On+H+M+H2On+H+→M+H++n  H2O

LTPI was already successfully applied for measurements under ambient conditions with superior performance. Moreover, it was also already used with a handheld low-temperature plasma source [99] and later for on-site analysis in combination with a miniature backpack mass spectrometer [100]. With this technology, explosives could be detected very quickly and in low quantities under ambient conditions from any surface [118] and even in mixtures [119]. Other promising applications were the screening of drugs of abuse [120, 121], agrochemicals in foods [122, 123], or fungicides in wine [124].

### 3.2. Relative Response with Low Temperature Plasma Ionization

In LTPI, analytes are typically detected as [M + H]^+^, it is a relatively soft ionization method with nearly no fragmentation of the analytes. In contrast to the still more common electrospray ionization [95, 125], sensitivity with the different plasma-based techniques or in dependence on source parameters was hardly investigated yet with the exception of the used plasma gas, electrode spacing and the power consumption related to the distances of the electrodes [18, 126–128]. With respect to compound characteristics, a low vaporization enthalpy and low polarity (i.e., log P, large molecular nonpolar surface area and the molar volume) of the analyte as the most influential factors in LTPI are advantageous for achieving high signal intensities [129]. In addition, for substances with a boiling point beyond 200°C, the supply of additional energy, e.g., in form of heat, might be recommended in order to achieve improved signal intensities [117, 129]. In general, a lower vaporization enthalpy results in easier evaporation and thus, the number of desorbed analyte molecules available for ionization in the gas phase is enhanced. Indeed, the use of higher temperatures during LTPI has already been described in the literature to improve analytical sensitivity [121, 122, 130, 131]. While ionization of low-boiling and less polar substances is particularly favoured, signal responses show a negative linear correlation to surface tension [129] which in return strongly correlates with the vaporization enthalpy in an inverse manner [132, 133].

**Figure 7:**
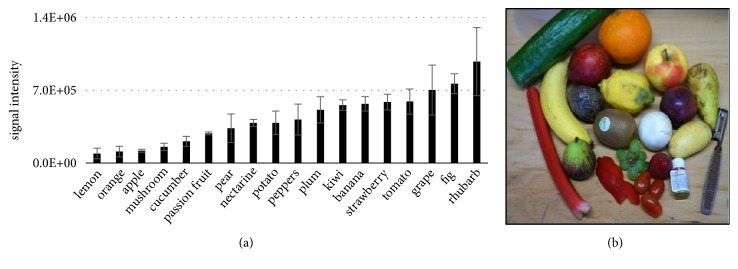
Signal intensity of LTPI measurements of chlorpyrifos as a function of the analyzed surface.

Compared to the impact of the analyte's molecular characteristics such as volatility and polarity and in contrast to ESI, the solvent exerted much less impact with respect to relative and absolute signal intensity. In general, a better signal intensity of the analyte was obtained with higher boiling solvents; however, except water, most of the solvents appeared to be almost equally suitable when using LTPI for MS [129]. Nevertheless, signal response in different solvents also seemed to be determined rather by specific interactions between analytes and solvent, indicating that unpredictable matrix effects will interfere with signal response in applications of this technique. Indeed, our LTPI analysis of chlorpyrifos from the surface of several fruits suggested the occurrence of such matrix effects modulating signal response by more than two orders of magnitude (Figure 7).

Surprisingly and at a first glance in contrast to conclusions made by Kiontke et al. [129], who suggested a favorable matrix for detection of aromatic amines featuring a relatively low vapour pressure with low surface tension, it might be concluded that the waxy surface consisting of high boiling constituents as present in citrus fruits makes a particularly bad matrix for sensitive detection of chlorpyrifos. Thus, detailed investigations of the observed effect in the range of a magnitude are required to better understand the reasons for such behavior. Possibly, the abundant presence of other volatile compounds on the surface of citrus fruits might create a transient microenvironment (TME, [134]) responsible for the observed effect decreasing LTPI efficiency. Specific analyte-matrix effects as well as a high variation in replicate analyses still seem to hamper quantitative analyses and further investigation is required to address this bottleneck. 

## 4. Differential Sensitivity of ESI, APCI and LTPI for the Aromatic Amines 

### 4.1. Influence of Compound Solution Basicity

The relative response of the aromatic amino compounds was finally used to further explore differential relative sampling efficiencies with the three atmospheric pressure methods, namely, LTPI [129], APCI, and ESI [15]. For that, correlation analysis of peak signal intensities with physicochemical properties was performed and compared.

In accordance to the literature, the results suggest that only the ESI response from a solution pH = 7 correlates well with the pKa of the analyte (R = 0.51, p < 0.01) (Figure S1a in the Supplementary Material). In ESI for the used set of aromatic nitrogen-containing compounds, the protonation of the analyte and subsequent desorption from the droplet is essential and at pH 7 (without additives) the signal intensity primarily follows the solution basicity of the analytes, where basic analytes can easily take up a proton from the solvent and desorb from the droplet. Less basic analytes are more difficult to protonate and therefore show a lower signal intensity. The situation is different after solvent acidification, where protonation is no longer the limiting factor due to an excess of protons in solution and the analytes are protonated more easily. Thus for ESI pH = 3, but also with APCI and LTPI (Figure S1b-d) no dependence of signal response on solution basicity was found. Moreover, also no correlation of the signal intensity with the proton affinity was found, which is somewhat surprising. According to the current understanding of APCI and LTPI ionization mechanism, the neutral analyte is vaporized and protonated solvent species transfer a proton to the amines in the gas phase depending on their proton affinity, which in turn corresponds to the acidity or basicity of a compound in the gas phase. Given that all of our analytes are nitrogen-bases with a gas-phase basicity > 830 kJ/mol providing a high proton affinity (as discussed before), other factors appear to be responsible for the observed sensitivity under the respective conditions.

### 4.2. Influence of Compound Volatility

**Figure 8:**
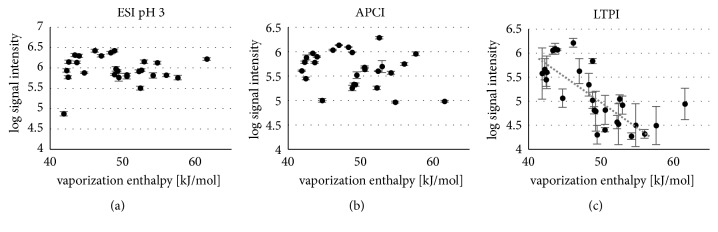
Log signal intensity (peak height) in dependency on the vaporization enthalpy for (a) ESI pH = 3, (b) APCI, and (c) LTPI (reprinted from [129] with permission by Springer 2018).

Among the volatility descriptors, boiling point, vaporization enthalpy, vapor pressure, and surface tension all were tested for their influence on relative signal response. Since the effect of the vaporization enthalpy was most prominent with LTPI, the obtained relative signal intensities for the three different ionization techniques were plotted over this parameter (Figure 8).

The most eye-catching observation is the high variance of LTPI response compared to the other two methods. An average standard deviation of 46% for plasma ionization (Figure 8(c)) was observed over all analytes, compared to 19% for ESI at pH 7 (Figure S2a) and 9% for ESI at pH 3 (Figure 8(a)) and APCI (Figure 8(b)), respectively. ESI and APCI are carried out in standardized, closed devices while the used plasma source has an open structure (Figure 6) and might therefore be more susceptible to variable environmental conditions such as convection, temperature, or humidity.

In accordance with the literature [131], for LTPI signal intensity a strong dependence on the analytes volatility was found based on negative correlation with the vaporization enthalpy (Figure 8(c), R = -0.64, p < 0.001), boiling point (not shown, R = -0.63, p < 0.001), and a moderate positive correlation with the vapour pressure (not shown, R = 0.55, p < 0.01). In general, a lower vaporization enthalpy improves the evaporation of the analytes making a larger number of analyte molecules amenable to ionization in the gas phase. In conclusion, this means that the supply of heat and the resulting improved evaporation and desorption of the analyte will result in a higher signal intensity. This behaviour has already been described in the literature to improve LODs with LTPI [121, 122, 130, 131]. However, no such correlation was observed for the other techniques except a positive correlation of signal enhancement in ESI response at pH 3 compared to pH 7 with the boiling point [15] which was also suggested to be a consequence of better desorption of more volatile compounds at adequate availability of charge carriers in solution, i.e., protons.

Furthermore, a very strong negative correlation (R = -0.86, p < 0.001) with the surface tension of the analytes was observed in LTPI (not shown). Surface tension is the result of increased cohesive intermolecular forces between molecules at the interfaces between air and liquid. For many liquids, the evaporation enthalpy changes linearly with the macroscopic surface tension [132, 133], and therefore both are dependent on each other. High surface tension of the analyte can suppress desorption and thus lead to a poor signal intensity. The fact that only LTPI is dependent on the volatility of the analytes tested here might be reasoned by the different temperature conditions applied during ionization; with APCI, sufficient energy was supplied by elevated temperatures through heated nitrogen streams (heater/auxiliary and dry gas at 250°C) to ensure complete vaporization.

**Figure 9:**
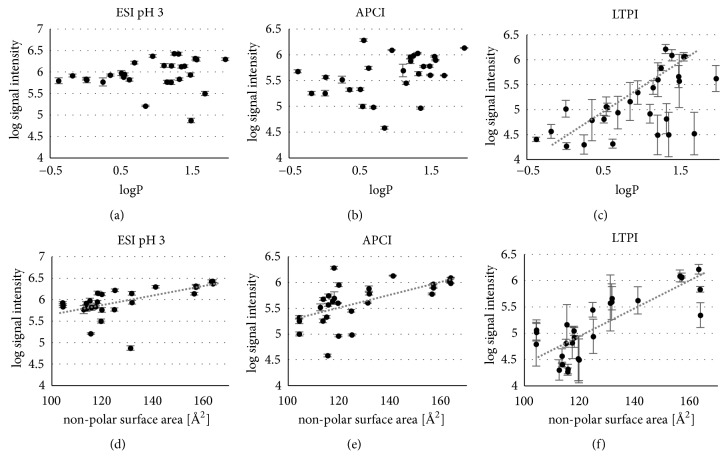
Log signal intensity (peak height) in dependency on the partition coefficient log P (SciFinder) for (a) ESI pH = 3, (b) APCI and (c) LTPI and the nonpolar surface area for (d) ESI pH = 3, (e) APCI, and (f) LTPI ((c) and (f) reprinted with permission from Springer [129] 2018).

While in ESI at pH 7 the influence of vaporization enthalpy seems overshadowed by the strong quantitative impact of solution basicity, at pH 3, when solution basicity loses its importance due to the enhanced availability of charge carriers, it is a low vaporization enthalpy leading to increased signal enhancement. However, at pH 3 other parameters than at pH 7 become important, in which impact was observed to be dependent on the particular instrument used, i.e., polarity or molecular size [15]. Thus, the influence of polarity was assessed for the three techniques; the results are illustrated in Figure 9.

Obviously, polarity of the analyte is a common parameter influencing the sampling efficiency of all three methods. Thus, for LTPI a strong correlation was found between the signal intensity and the log P (data from ChemAxon, Figure 9(c), R = 0.61, and p < 0.001) and an even very strong correlation with the nonpolar surface area (Figure 9(f), R = 0.81, and p < 0.001). The signal intensity in APCI also shows a moderate correlation with the log P (R = 0.48, p < 0.01, and Figure 9(b)). Interestingly, for ESI at pH 7 polar compounds show an increased sampling efficiency (Figure S2b and c), while for APCI and LTPI the reversed behaviour was observed. This hints to the interplay between ionization and desorption; in ESI, ionization occurs* before* (ion) desorption, while for APCI and LTPI desorption is first and the desorbed analyte is* afterwards* ionized in the gas phase; thus, desorption of the* neutral* analyte would benefit from a low polarity favouring the droplets liquid/gas interface while for ESI, where chargeability* in solution* has the highest impact on sampling efficiency, a higher polarity is beneficial. Disappearance of this influence in ESI at pH 3, where chargeability is largely improved by the enhanced density of charge carrier, would support this perception.

A similar situation may apply to the size of the nonpolar surface area. In situations, where desorption is not limited by a required charge; i.e., for ESI at pH = 3 (Figure 9(a), R = 0.56, and p < 0.01) and APCI (Figure 9(b), R = 0.59, p < 0.01, a moderate correlation), a correlation of this parameter with the sampling efficiency was observed. Both descriptors, polarity and nonpolar surface area, have an impact on the surface activity of an analyte [23, 80] and help to improve desorption, which is necessary for a good signal intensity. Ions and molecules with a large nonpolar area have a high surface affinity, as they prefer the air-liquid interface before the aqueous bulk solution within the droplets.

**Figure 10:**
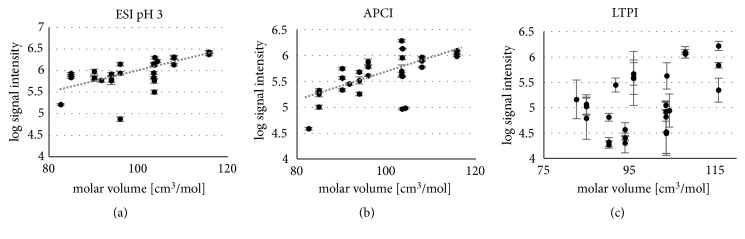
Log signal intensity (peak height) in dependency on the molar volume for (a) ESI pH = 3, (b) APCI, and (c) LTPI (reprinted with permission from Springer [129] 2018.)

Finally, Figure 10 illustrates the influence of the molar volume on the signal intensity obtained with the investigated ionization techniques.

Within this context, the strong correlation of the molar volume with the signal intensities of ESI pH = 3 (Figure 10(a), R = 0.61, and p < 0.001), APCI (Figure 10(b), R = 0.64, and p < 0.001) and a moderate correlation for LTPI (Figure 10(c), R = 0.49 p < 0.01) also fits in well. In ESI, the competition for charge and desorption takes place on the surface of the droplet, but only charged species will be further transmitted. Furthermore, it is known that a (charged) analyte in ESI needs access to the droplet's surface for successful detection [135]. Again, at pH 7 the limited availability of charge carriers overshadows the impact of the molar volume (Figure S2d), but at pH = 3 it can be assumed that all analyte molecules are protonated in the droplet. The charged particles tend to spread over the surface of the droplets at maximal distance due to Coulombs repulsion. According to Wu et al., ions with larger molecular volumes can occupy a larger proportion of the droplet surface than ions with smaller volumes [136] enabling easier desorption from the droplet (note that the molecular volume strongly correlates with the molar volume). Furthermore, the larger the molecule, the better its protonated form is stabilized in the gas phase [81] which should exert its effect in all three, ESI, APCI, and plasma ionization.

Under the chosen conditions, desorption from the solvent seems to play a similarly strong role for LTPI and APCI as solution basicity for ESI at pH 7. This was initially unexpected for APCI, as complete evaporation should be ensured with the help of nebulizer, heated auxiliary gas and dry gas in accordance with the APCI mechanism. A better evaporation (choosing a higher temperature for the APCI heater and the dry gas, or an easier vaporizable solvent) may counter the observed dependence and uncover other influencing variables than the nonpolar surface area or the molar volume. Desorption and thus surface activity is also one of the key aspects in LTPI [129]. As no additional heat was added, the analytes have to first reach the surface of the solvent droplet and then evaporate. In this case, the nonpolar surface of a species is decisive for the deposition on the droplets surface, as is the vapour pressure, respectively the boiling temperature for the subsequent evaporation. However, in LTPI still a droplet-pick up mechanism seems to crucially enhance the sampling efficiency since the mere presence of a solvent was observed to enhance signal response significantly [129].

**Figure 11:**
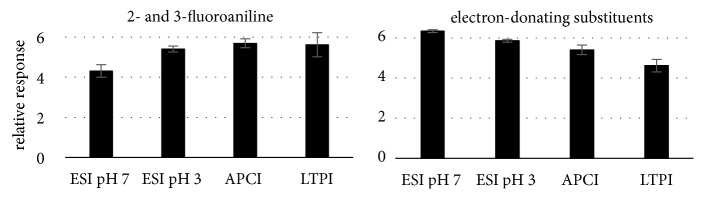
Response pattern of 2- and 3-fluoroanilines vs. electron-donating substituted compounds over the four ionization conditions reflect the fact that ESI pH 7 response is dominated by solution basicity while this advantage is lost when it comes to conditions where volatility of the compound matters.

The detailed investigation on the quantitatively most impacting parameters of the three ionization techniques resulted in a rather complex and sophisticated picture which prompted us to analyze similarities in the pattern of obtained signal responses within the three ionization techniques. As a result, response pattern after ESI at pH 3 appeared to be almost equally similar to all other conditions, i.e., ESI pH 7 (R = 0.54, p = 0.003), APCI (R = 0.51, p = 0.001), and LTPI (R = 0.65, p < 0.001), which suggests that solvent acidification in ESI leads to a situation where sensitivity becomes dependent on similar analyte characteristics as in APCI and LTPI instead of the solution basicity. However, the correlation between the two ESI conditions greatly improved using the signal log values for analysis (R = 0.75, p < 0.001) but impaired the cobehaviour with APCI and LTPI. After log-transformation, the influence of extreme values in a linear correlation analysis is usually decreased. Therefore, we concluded that similarity of response patterns in ESI pH 3 with pH 7 might be based on the behaviour of different analytes than similarity with APCI and LTPI and analysed the cobehaviour of the target compounds. Indeed, we found a reverse response pattern of analytes with amino-, hydroxyl-, and methoxy-substituents as one group and a second comprising mainly pyridine, 2- and 3-fluoroanilines, and analytes with electron-withdrawing substituents in* o*-position (Figure 11).

While for the target compounds with electron-donating substituents, the highest relative intensity was observed with ESI pH 7 where solution basicity determines the response, the behavior is reversed for volatile analytes with electron-withdrawing and less polar analytes due to H-sharing of the substituent in* o*-position to the amino group [15]. Consequently, when we removed 2- and 3-fluoroaniline and pyridine, where this appearance was most developed, from correlation analysis of the log transformed data, we again obtained the strongest correlation between ESI pH 3 and LTPI instead (R = 0.70, p < 0.001 vs. R = 0.49, and p = 0.014 for ESI pH 3/ pH 7). Consequently, requirements for a good ESI response of polar, strong bases in acidified solvent are most similar to those in LTPI, while less polar weaker bases exhibit an impaired response in ESI pH 7 compared to the other analytes and ionization techniques leading to an (unwanted?) selectivity of ESI.

Interestingly, a comparison of the correlation factors obtained from analysis of response in relation to all molecular descriptors indicated the highest similarity of influencing factors for ESI pH 3 and APCI. Obviously, however, the quantitative impact of these molecular descriptors on signal response is different under the two conditions given the results detailed above.

## 5. Conclusions

In our comparison of two standard atmospheric pressure techniques, ESI and APCI, with the still quite new, ambient LTPI, we could show that generally different parameters of desorption properties, determined by the descriptors of volatility and surface affinity, seem to be decisive for the signal response of an analyte in a liquid matrix. Only at ESI pH 7 does the influence of basicity exceed the influence of desorption properties (Table 1).

According to this, APCI seems to be the least selective method against the aromatic amines, which would be a great advantage when it comes to multiselective analysis or screening methods. However, within this context the dynamic range of signal response (highest divided by least abundance) with ESI pH 3 actually suggested a weaker selectivity (factor 35 for ESI pH 3 vs. 50 for APCI contrasting ~700 for ESI pH 7) and indicates that it is rather the fact that, in APCI a more complicated, but not too strong affecting interplay of compound characteristic, might determine its signal response than nonselective ionization. The partially large differences in the quantitative impact of the various molecular descriptors between the methods also illustrate the fact that there is still no universally optimal ionization method available for all analytical tasks. The requirement of ionization and subsequent transfer of the ion into the gas phase still is the main limitation in application of MS. Therefore, at present and for the foreseeable future, it is only possible to optimize the interface for a specific analytical challenge and a multitude of further developments may still be required towards an optimal, generally applicable ionization method for MS.

## 6. Materials and Methods

### 6.1. Chemicals

3-aminophenol, 2-fluoroaniline, 3-fluoroaniline, 4-fluoroaniline, 2-methoxyaniline (*o*-anisidine), 3-methoxyaniline (*m*-anisidine), 4-methoxyaniline (*p*-anisidine), 2-nitroaniline, 3-nitroaniline, 4-nitroaniline, 3-methylaniline (*m*-toluidine), 3-aminoaniline (*m*-phenylenediamine), 4-aminoaniline (*p*-phenylenediamine), 2-aminobenzonitrile, 3-aminobenzonitrile, 4-aminobenzonitrile, and pyridine were purchased from Sigma Aldrich (Taufkirchen, Germany), besides 2-methylaniline (*o*-toluidine), 4-methylaniline (*p*-toluidine), chlorpyrifos from Fluka (Buchs, Switzerland), and aniline from Acros (Geel, Belgium). Acetonitrile (ACN) was purchased from VWR (Dresden, Germany). Methanol was purchased from Carl Roth (Karlsruhe, Germany), water from BIOSOLVE (Valkenswaard, Netherlands). 2-aminoaniline (*o*-phenylenediamine), 2-aminopyridine, 3-aminopyridine, 4-aminopyridine, 2-aminophenol, 4-aminophenol, 2-aminobenzoic acid, 3-aminobenzoic acid, 4-aminobenzoic acid, sulfanilic acid, and 4-chloroaniline were kindly provided by Professor* em.* S. Berger (Institute of Analytical Chemistry, University of Leipzig, Germany).

### 6.2. Sample Preparation

A set of 31 anilines was prepared in ACN/H_2_O 1:1 (*v/v*) for each aniline (10 *μ*M for ESI and APCI, 1 mM for LTPI) and the signal intensity was determined with each of the three ionization methods, namely, atmospheric pressure chemical ionization (APCI), electrospray ionization (ESI), and low temperature plasma ionization (LTPI). Solvent blanks were run on a frequent basis to ensure the absence of cross-contamination. Prior to the measurement of the complete set of aromatic amines, dynamic behaviour was successfully confirmed using concentration series of the analytes [15, 129]. For detailed structures of all analytes see Kiontke et al. [129]. The analytes were selected for a systematic study of the influence of the molecular descriptors such as polarity and vapour pressure; they provide a very broad structural variety, importance in biological contexts and many compounds of interest contain structural units that are similar to our analytes. In addition, these analytes are already well characterized in the literature and publicly available databases.

#### 6.2.1. APCI Response of Anilines

APCI measurements were performed on a Bruker Impact II QTOF MS (Bruker, Bremen, Germany) equipped with a Dionex Ultimate 3000 autosampler (Thermofisher, Dreieich Germany). The capillary voltage was at 4.0 kV, and 100 *μ*L sample was injected at a flow rate of 50 *μ*L/min with 2.5 bar nebulizer and 2.5 L/min dry gas flow rate (both nitrogen) at 250°C. Scan range was* m/z* 50-500 with 3 scans rolling average.

#### 6.2.2. LTPI Response of Chlorpyrifos

To simulate the on-site analysis of pesticides directly from the surface using plasma ionization, fruit, vegetables and mushrooms were purchased from a local supermarket (REWE, Tarostraße, Leipzig, Germany). A strip of apple, banana, pear, mushroom, strawberry, fig, cucumber, potato, kiwi, nectarine, orange, pepper, passion fruit, plum, rhubarb, tomato, grape, and lemon peelers at least 1 cm long was taken from each peel to obtain the most reproducible layer thickness. Four circular cut-outs with a diameter of 4 mm were punched out of each of these peel strips. Subsequently, 1 *μ*L chlorpyrifos solution (100 *μ*M in ACN) was added to three cut-outs (the fourth served as blank) and the solvent was allowed to evaporate before the measurement.

An optimized home-built low-temperature plasma source was used in the experiments [18]. Briefly, it consists of an ignition transformer (EBI4 CM S, Danfoss, Nordborg, Denmark) and a glass tube (GC liner, Thermo Scientific, Waltham, MA, USA) with two surrounding outer electrodes made of copper foil tape (Noll GmbH, Wörrstadt, Germany) and isolated by a homemade Teflon housing. The flow was adjusted to 20 mL/min with an Ellutia 7000 GC Flowmeter (Ellutia Ltd, Ely, UK).

Optimized parameters of plasma configuration were used [18], e.g., dielectric thickness 2 mm, width and distance of the electrodes 10 mm each and distance of the electrode to the outlet 20 mm. Mass spectra were acquired on an Esquire 3000+ MS (Bruker, Bremen, Germany) with the following instrumental parameters: high voltage off, dry gas (nitrogen) 1.5 mL/min with a temperature set to 350, scan range:* m/z* 50-400, target mass: 350. The number of ions per scan was limited to 20,000 with a maximum accumulation time of 200 ms and a rolling average of three scans. After spotting 1 *μ*L of the solutions on a paper target, data acquisition was immediately started at least for 2 minutes and the average response of each analyte was calculated from triplicate analysis.

### 6.3. Data Evaluation

The* m/z* peak signal intensities were averaged over 1 min analysis time using Bruker Data analysis software 4.2 and the corresponding signal intensities of triplicate analyses were used for data evaluation. The relative response of the anilines was assessed as the average intensity (cps, peak height) of the corresponding peak for the [M + H]^+^ ion of the analyte of interest and for chloroaniline as sum of the two most abundant isotope peak signal intensities.

Characteristic chemical constants (pKa, molecular polar surface area, solvent accessible molecular surface area, log P, log D, proton affinity, gas phase basicity, boiling point, vapour pressure, vaporization enthalpy, and surface tension) were retrieved from public databases, namely, ChemSpider by the Royal Society of Chemistry, London, UK [http://www.chemspider.com/], chemicalize.org by ChemAxon, Budapest, Hungary [http://www.chemicalize.org/], Scifinder by the Chemical Abstracts Service, Columbus/Ohio, USA [https://scifinder.cas.org/], and the NIST Chemistry WebBook by the National Institute of Standards and Technology (NIST), Gaithersburg, USA [http://webbook.nist.gov/chemistry/]. The molecular volume was calculated using the Spartan software package (Spartan 14, Wavefunction Inc., Irvine, CA, USA). The settings for calculation were DFT (density functional theory) B3LYP with a 6–31G*∗* basis set.

Correlation analysis of peak signal intensities with physicochemical characteristics (Pearson's product-moment correlation coefficient and significance) was carried out using the Analysis ToolPak in MS Excel 2013 (Microsoft Corp., Redmond, USA). Before correlation analysis, a visual inspection of appropriate data distribution was carried out using scatter plots.

## Figures and Tables

**Table 1:** Summary of analyte and solvent characteristics in how they impact sampling efficiency of the three ionization methods, ESI, APCI, and LTPI, for MS.

	ESI pH 7	ESI pH 3	APCI	LTPI
*Analyte*				
surface tension	(disadvantageous)	disadvantageous	no influence	disadvantageous
polarity	beneficial	(beneficial)	disadvantageous	disadvantageous
volatility	no influence	beneficial	no influence	beneficial
basicity	beneficial	(beneficial)	no influence	no influence
fragmentation	hardly observed	hardly observed	observed	hardly observed
molar size	beneficial	beneficial	beneficial	beneficial

*Solvent*				
surface tension	disadvantageous	disadvantageous	not analyzed	disadvantageous
polarity	beneficial	no influence	not analyzed	no influence
volatility	beneficial	beneficial	not analyzed	disadvantageous
pH	contradictory*∗*	contradictory*∗*	not analyzed	not analyzed

Reproducibility	moderate	high	high	low

*∗*Depending on the interaction between solvent evaporability, electrolyte, and instrumental configuration [15].

## Data Availability

The data from ESI-MS and LTPI-MS analyses (nitrogen-containing aromatic compounds) supporting this report are from previously reported studies and datasets, which have been cited. The data on APCI-MS (nitrogen-containing aromatic compounds) and LTPI-MS chlorpyrifos analyses used to support the findings of this study are available from the corresponding author upon request.

## Supplementary Materials

Supplementary Materials Supplementary material associated with this report can be found online (Figures S1 a-d and Figures S2 a-d).
