# Supplementary material for: Response in Ambient Low Temperature Plasma Ionization Compared to Electrospray and Atmospheric Pressure Chemical Ionization for Mass Spectrometry
Source: Int J Anal Chem. 2018 Dec 18;2018:5647536. doi: 10.1155/2018/5647536 (PMC6339754; doi:10.1155/2018/5647536)
Supplement: Supplementary Materials — Supplementary material associated with this report can be found online (Figures S1 a-d and Figures S2 a-d). [file 5647536.f1.docx]

Supplementary Materials

Response in ambient low temperature plasma ionization compared to electrospray and atmospheric pressure chemical ionization for mass spectrometry

Andreas Kiontke, Susan Billig and Claudia Birkemeyer*

Research Group of Mass Spectrometry at the Faculty of Chemistry and Mineralogy, University of Leipzig, Linnéstr. 3, 04103 Leipzig, Germany

*Correspondence should be addressed to Claudia Birkemeyer; [birkemeyer@chemie.uni-leipzig.de](mailto:birkemeyer@chemie.uni-leipzig.de)


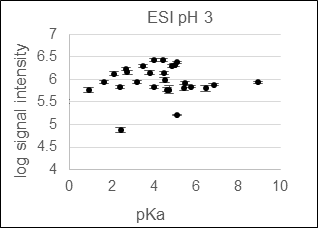

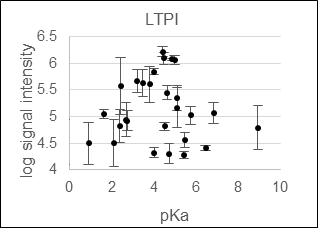

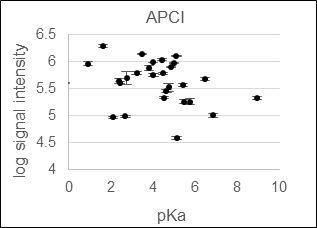

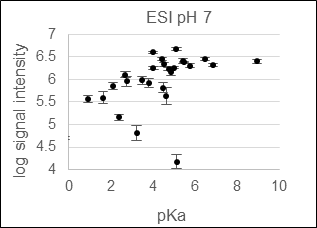


a

b

c

d

**Fig. S1** *Log signal intensity (peak height) of the aromatic amines in dependency on the pKa (ChemAxon) for* ***(a)*** *ESI pH 7,* ***(b)*** *ESI pH 3,* ***(c)*** *APCI and* ***(d)*** *LTPI.*


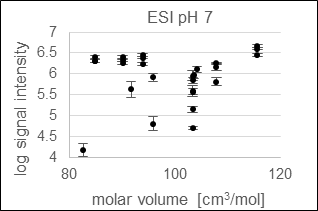

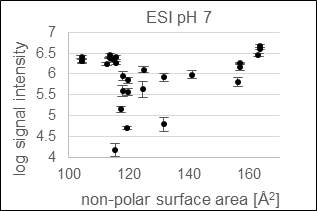

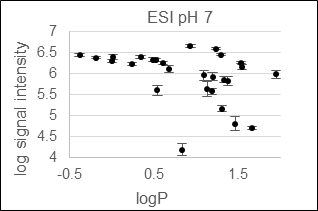

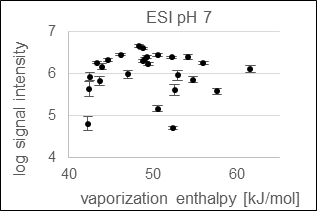


a

b

c

d

**Fig. S2** *ESI pH 7* *log signal intensity (peak height) in dependency on the* ***(a)*** *vaporization enthalpy,* ***(b)*** *logP (Scifinder),* ***(c)*** *nonpolar surface area and* ***(d)*** *molar volume.*
